# Supplementary material for: Mesoscale structure development reveals when a silkworm silk is spun
Source: Nat Commun. 2021 Jun 17;12:3711. doi: 10.1038/s41467-021-23960-w (PMC8211695; doi:10.1038/s41467-021-23960-w)
Supplement: Supplementary file 1 — Supplementary Information [file 41467_2021_23960_MOESM1_ESM.docx]

*Supplementary information*

**Mesoscale structure development reveals when silk is spun**

Quan Wan^1^, Mei Yang^1^, Jiaqi Hu^1^, Fang Lei^1^, Yajun Shuai^1^, Jie Wang^1^, Chris Holland*^2^, Cornelia Rodenburg*^2^, Mingying Yang*^1^

^1^ College of Animal Science, Zhejiang University, China

^2^ Department of Material Science and Engineering, University of Sheffield, UK

1. Mechanical properties of prepared samples

The mechanical properties of samples that reached different stages can be seen below (supplementary information Table 1). A sample’s progression stage was confirmed by the morphology of the failure cross sections.

Supplementary Table 1: The mechanical properties of drawn samples in each progression stage

| Sample No. | 1 | 2 | 3 | 4 | 5 | 6 | 7 | 8 | 9 | 10 | 11 | 12 | 13 | 14 | 15 | 16 | 17 |
| --- | --- | --- | --- | --- | --- | --- | --- | --- | --- | --- | --- | --- | --- | --- | --- | --- | --- |
| Tensile strength (MPa) | 18 | 48 | 30.7 | 20.3 | 69 | 176 | 195 | 123 | 86 | 165 | 60 | 463 | 417 | 482 | 381 | 294 | 203 |
| Young’s modulus (GPa) | 0.46 | 1.2 | 0.42 | 0.2 | 1.1 | 4.4 | 3.9 | 0.73 | 0.64 | 2.29 | 0.4 | 8.76 | 9.78 | 9.67 | 4.22 | 3.25 | 3.49 |
| Maximum strain (%) | 4.3 | 6.7 | 7.2 | 10 | 11 | 6.9 | 10.6 | 16.9 | 16 | 15.3 | 19.3 | 32 | 29.9 | 14.8 | 15.7 | 24.1 | 8.4 |
| Toughness (MJ/m^3^) | 0.27 | 2.27 | 0.76 | 0.72 | 4.77 | 10.5 | 9.27 | 8.45 | 6.84 | 7.38 | 5.4 | 70.3 | 67.2 | 35.2 | 32.9 | 57.3 | 10.3 |

*The sample 7-9 and 13-15 are measured from drawn samples, the sample 10-11 and 16-17 are extruded samples, sample 6 and 12 is a fibrillization stage sample before and after post draw.

**Sample 1-5 are arrested in gelation stage, sample 6-11 in fibrillization stage, sample 12-17 in consolidation stage.

1. Surface Fibril Patterns and water pocket fusion

The development of nanofibrils in the drawn samples in section below is identical to the discussion in manuscript Fig. 2 and Fig. 3. Supplementary information Fig. 1 (a-d) shows the gel to nanofibril progression on a sample surface with increased strain accumulation from supplementary information Fig. 1 (a) to (d). This is a pattern created by the accumulation of strain and as sign of nanofibril formation. The size of nanofibrils decrease as strain accumulates, and reach around 40nm diameter as sample reaches the consolidation stage.

Water pockets only appear close to the surface at the very peak of the water release process where their density is highest and overall pockets are rarely visible at the sample surface, presumably due to their rapid release and dissipation. Generally, the size of the sub-surface water pocket observed in our samples varies from 70nm to 200nm diameter. We speculate that the size depends on local dehydration rate, which depends on local crystallization as crystallized regions hinder the escape of water since water cannot easily pass through such dense regions.


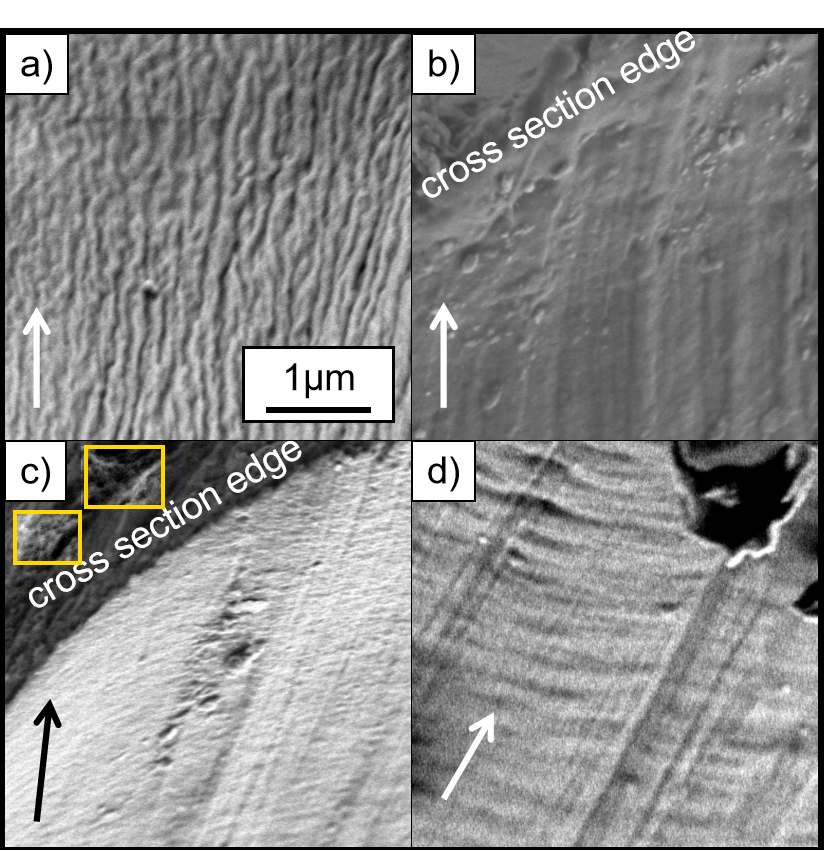


Supplementary Figure 1: SEM images of the surface of drawn solution-gel samples are shown from low strain to high strain in the order of a, b, c, d. The image (d) is close to complete fibrillization (calculated accumulated extensional strain ~2). The strain axis is shown by arrows. Note the pockets in highlighted area in image (c). The field of view of all SEM images above is 3.5×3.5μm^2^, and their contrast (Secondary Electron images) represent topographical feature.

1. Pultrusion and extrusion spinning

Fibre spinning parameters were decided by monitoring the mechanical performance in the drawing or spinning process. As shown in the main manuscript, the spinning process consist of assembling and consolidation of the silk protein, thus any sample ready for producing a fibre must exhibit: (1) a low level of chain folding and crystallization, allowing for the potential for inter-molecular and inter-phase interaction and (2) a suitable level of protein concentration and chain entanglement, where proteins are close enough to interact easily with each other. While these conditions are not directly measurable, such properties manifested themselves as a simple drawing behaviors in our experiments: i.e. the drawing force will reach a plateau value when using uniform drawing speed with respective drawing stress of several MPa (supplementary information Fig. 2 a).

Samples for which a plateau stress is observed during drawing follow the structure progression discussed in the manuscript. The whole process from post drawing to stress failure of an example sample is shown in plot supplementary information Fig. 2 (b), which yields a post drawing strain plateau at ~6MPa, followed by stress failure (low failure stress due to wet sample).

Drawn sample can reach a consolidated state at different drawing strain and speed and these parameters affected the final mechanical properties of the silk fiber. This is reflected in the varying strength and toughness value shown in supplementary information table 1. The difference of fiber diameter may also affect dehydration and thus cause varied strength, as reported in natural silk^[[1]](#endnote-0)^. However we have little control of these parameters in our experiment as they depend on the viscosity of system and thickness of fiber. Investigation of these parameters for improved silk spinning would be subject of future research.

The extrusion spun samples are largely defined by the die hence the spinning parameters of the extruded sample can only be selected from a few values. The stress requirement of extrusion follows a similar trend to pultrusion ones, that the higher spinning speed (thus higher accumulated strain) leads to a higher stress requirement. As shown in supplementary information Fig. 2 (c), the stable extrusion stress increased from 0.4MPa to >8MPa when the accumulated extensional strain increases from 1.7 to 2.4. Our extrusion spun samples used parameters within their respective stable extrusion regions.


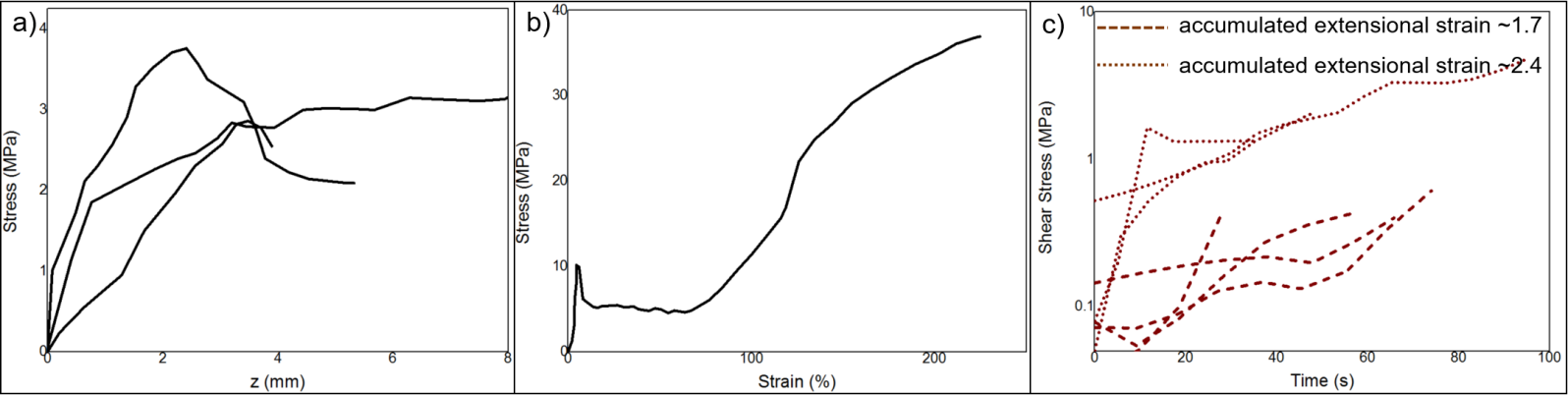


Supplementary Figure 2: Plot (a) shows the stress requirement for 2mm/min drawing process of several mm length of three sets of samples. Plot (b) is the stress-strain curve of a wet fibrilization stage drawn sample post drawn and until failure. Plot (c) shows the extrusion stress required to produce an extruded sample. Dashed plots represent samples with accumulated extensional strain of ~1.7 and dotted ones represent samples with accumulated extensional strain of ~2.4.

1. Simulation of extensional flow and calculation of accumulated extensional strain

The calculation of accumulated extensional strain is based on a reported model as mentioned in the main manuscript^[[2]](#endnote-1)^. There are two steps in calculating the required parameter for this model and an example is given below.

First, we draw the shape of our drawn sample in COMSOL software based on the image of sample as shown in the main manuscript in Fig.2. Since the scale of some sample segments are in tens of micrometers we simplify the outer boundary of the last few millimeters as a Bezier curve. The calculation model is selected as transient simulation of creeping flow. We have selected a non-Newtonian Carreau model based on literature, and the density of our sample is 1.14g/ml. The Carreau model applied in COMSOL software is shown in equation (1):

$\eta(\gamma)=\eta_{\inf}+(\eta_{0}-\eta_{\inf})[1+(\lambda\gamma)^{2}]^{\frac{n-1}{2}}$ (1)

*η* is dynamic viscosity

*γ* is shear rate

*η_0_* is zero shear viscosity

*η_inf_* is infinite shear viscosity

*n* is model constant

*λ* is relexation time

Since we do not have access to parameters of the viscosity model, we fitted an experimentally measured viscosity versus shear rate plot into the model equation using Origin 9 software as shown in supplementary information Fig 3 (a). The fitted equation parameters are *η_0_* = 5600 Pa*s, *n* = 0.14 and *λ* = 0.5s, fitted value of *η_inf_ is low and we approximate it at* 0 Pa*s.

The fitted parameters are applied in the COMSOL simulation, the inlet boundary is set according to the stress measured in our drawing process and outlet boundary is set to 0Pa. The velocity field is calculated as shown in the manuscript Fig. 4, and a series of data points on the central *z* axis every 0.5mm in the last 4mm was selected to calculate the extensional strain (supplementary information Fig. 4). The equation for accumulated extensional strain is shown as below in equation (2):

$\varepsilon(z)=\int_{0}^{z} \frac{dv_{centre}(z)}{v_{avg}(z)}$ (2)

*ε* is accumulated extensional strain

*z* is flow distance

*v_avg_* is the average velocity of chosen flow field cross-section

*v_centre_* is the velocity at centre of chosen flow field cross-section

In addition, the effective accumulated extensional strain is the calculated value above a critical extension rate, which indicates the onset of the polymer coil-to-stretch transition occurs. This value is approximately 0.5/*λ*, which is 1s^-1^ for our sample. The resulting accumulated strain is shown in manuscript Fig. 3.


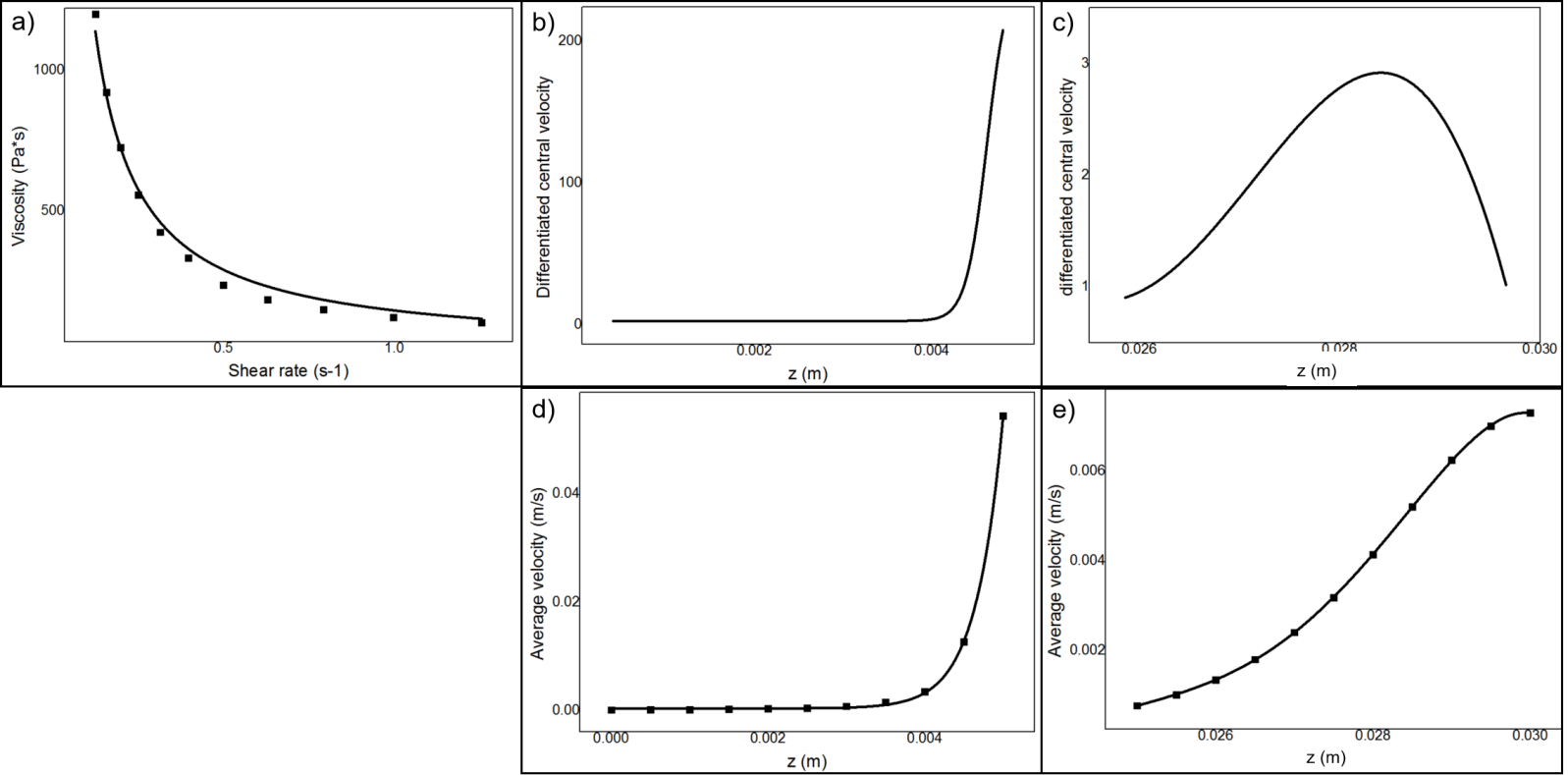


Figure 3: The plot (a) is the dynamic viscosity of our solution-gel data fitted using the Carreau model, the dots are measured data and the curve is fitted. Plots (b, c) are the calculated differentiated central velocity of pultrusional and extrusional spun sample using simulated flow field, and plots (d, e) are the average velocity data and their fitted equation for calculation of pultrusional and extrusional spun sample.

1. Composition and characterization of regenerated silk protein

Due to our mild degumming method, the composition of our regenerated protein is different from conventional ones. We’ve presented the SDS-PAGE result of our regenerated protein in supplementary information Fig. 4. The majority of our sample is concentrated in region over 120kDa, which correspond to the high-molecular mass parts of fiborin. A major band is shown at ~26kDa and a minor band is observed at ~32kDa in the low molecular mass region as well as a light band at ~66kDa. Light components such as P25, light chain of fibroin^[[3]](#endnote-2)^ and some part of sericin^[[4]](#endnote-3)^ is reported to be related with this molecular mass region. In summary, molecular mass data suggests our sample contain both lighter and heavier components of silk protein.

However considering the variation of regenerated silk protein molecules, we do not aim to approach native-like status through controlling molecular composition but rather through approaching the high viscosity of native silk dope. This is also due to the utility of different protein components in silk spinning is complicated^[[5]](#endnote-4),^^[[6]](#endnote-5),^^[[7]](#endnote-6)^. In brief, light molecular mass components (linkers and amorphous segments) of fibroin are playing an assistant role in fold and adhesion of core repeating units of beta-sheet crystal, and this system is stabilized by sericin in solution. However the proposed mechanism is not well established enough and detailed molecular behavior is still elusive, we cannot build a direct relationship between protein composition and structure development.

Thus we focus on the rheological parameter of our regenerated silk protein as a more practical approach. In our experiments, this regenerated protein system (25% wt) yield over 1000Pa*s at 0.1s^-1^ shear rate viscosity as shown in supplementary information Fig. 4 (c). In addition, a shear stress increase is observed at 1s^-1^ shear rate, indicating the start of shear-induced phase transition. These parameters are close to the value of natural silk^[[8]](#endnote-7)^, which indicates sufficiently long protein chain to maintain entanglement. And this contributed to the recurrence of natural shear-flow driven spinning and formation of hierarchical structure.


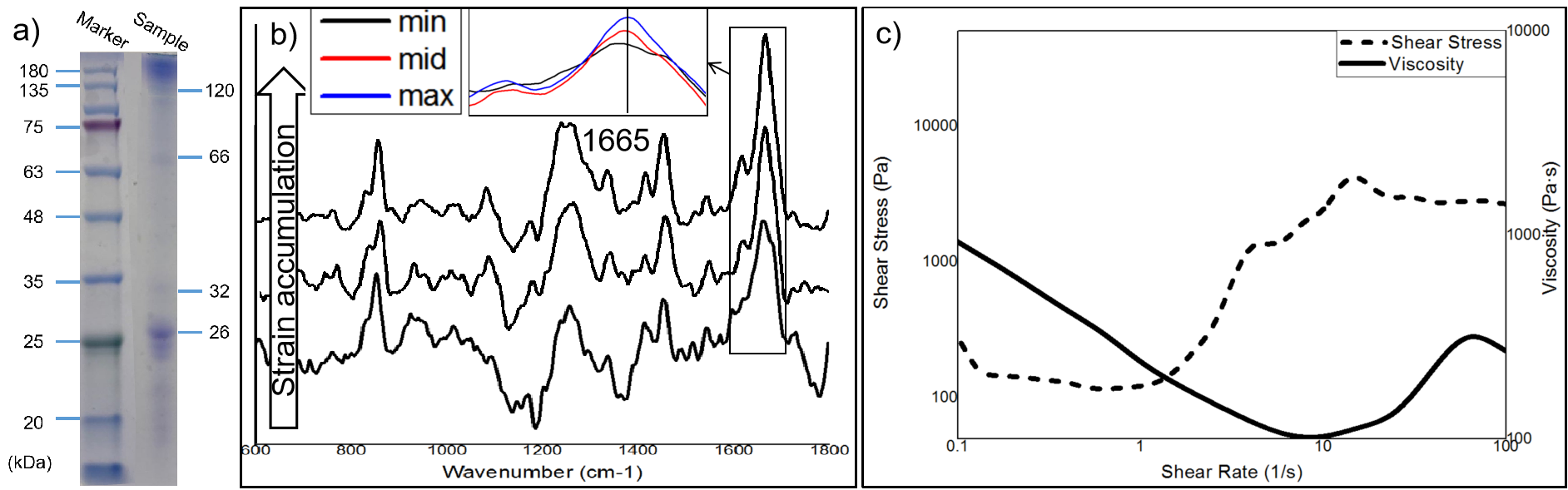


Supplementary Figure 4: Picture (a) is the SDS-PAGE result of our regenerated protein, left row is marker and right row is our sample, show several clear bands from 25 - 120kDa besides the large molecular mass components over 120kDa. Plot (b) showed Raman spectra of three samples in our spinning, with different strain accumulation. The increase of beta-sheet corresponding peak as strain accumulates is zoomed in in upper box. Plot (c) is the viscosity and shear stress measured for 25% wt concentration sample versus shear rate.

As the processing of our protein system followed natural spinning-drawing method, its molecular secondary structure shows similar trend in spinning and drawing. Supplementary information Fig. 4 showed the Raman signal alteration of our samples as accumulated extensional strain increases. The peak of beta-sheet at 1665cm^-1^ reflected the increase of crystallinity in fiber formation, however calculated fraction of beta-sheet content do not exceed 25% even after consolidation stage. On the other hand, an increase of hydrophobicity peak height at 850cm^-1^ suggested increase of hydrophobic group, thus occupation or folding of hydrophilic group. Such molecular crystallinity is similar to natural silk and much lower than many artificial silks. In the main manuscript, we described a major increase of fiber strength from ~200MPa to ~500MPa before and after consolidation stage, respectively. Our crystallinity measurement suggests not only molecular level structure, but also increase of nanoscale organization contributed to this increase.

1. Raw data applied in measurement of samples

The raw sample cross-section images used in measurement of volume fraction of water pockets and crystallized fibroin is shown below in supplementary information Fig. 5. And the raw mechanical testing strain-stress data is provided in supplementary information Fig. 6.


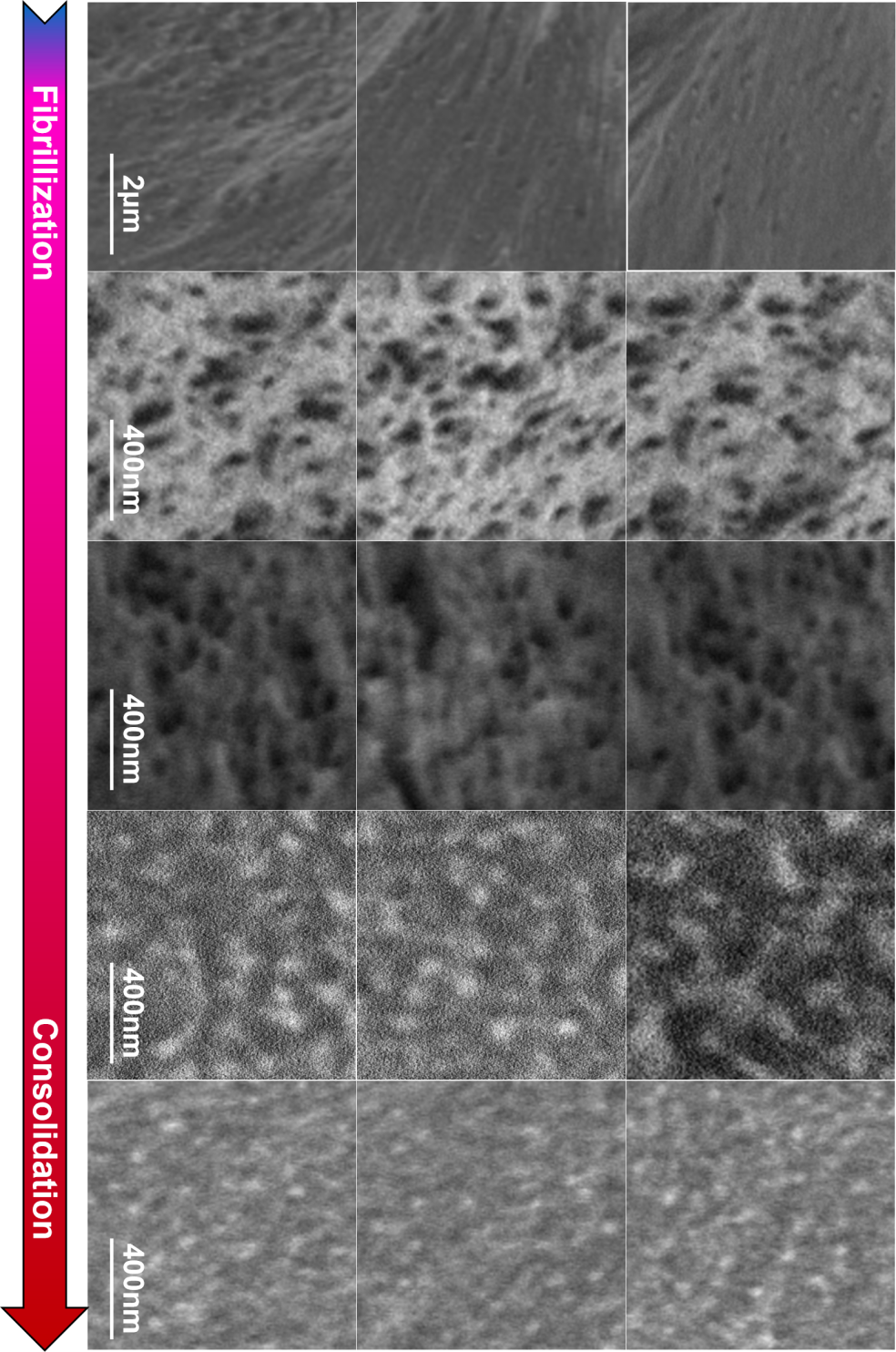


**e)**

**d)**

**c)**

**b)**

**a)**

Supplementary Figure 5: The raw cross-section SEM images of three sets of drawn samples is shown, each image column (a-e) represent one data point in the measurement of structure volume fraction of different stages. The image is collected with back scattering electron and brighter region represent higher density.


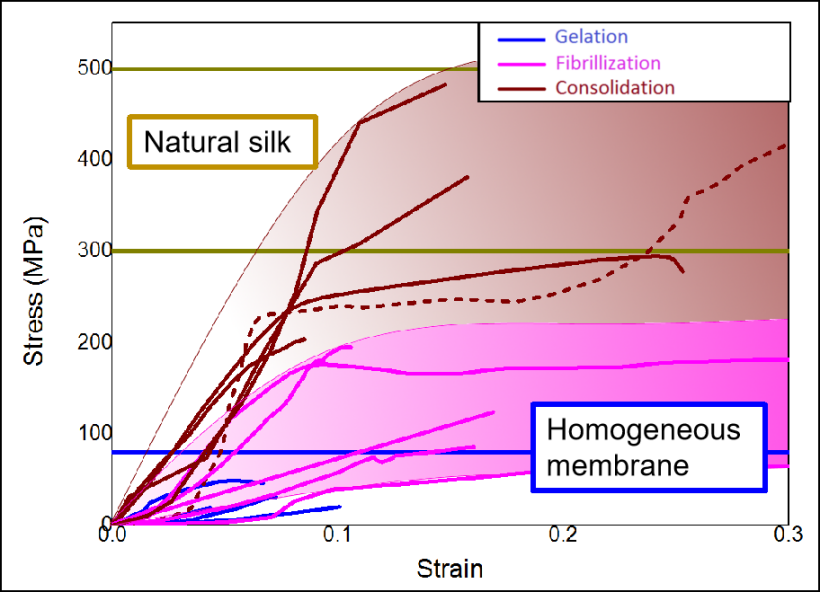


Supplementary Figure 6: The raw stress-strain testing data of samples in different stages is displayed with comparison to homogeneous silk fibroin membrane and natural silk fiber. Dashed line represent a sample reached consolidation through post drawing.

References

1. Chen, S., Liu, M., Huang, H., Cheng, L. & Zhao, H. P. Mechanical properties of Bombyx mori silkworm silk fibre and its corresponding silk fibroin filament: A comparative study. *Mater. Des.* **181**, 108077 (2019) [↑](#endnote-ref-0)
2. Breslauer, D. N., Lee, L. P. & Muller, S. J. Simulation of flow in the silk gland. *Biomacromolecules* **10**, 49-57 (2009) [↑](#endnote-ref-1)
3. Laity, P. R., Baldwin, E. & Holland, C. Changes in Silk Feedstock Rheology during Cocoon Construction: The Role of Calcium and Potassium Ions. *Macromol. Biosci.* **19**, 1800188 (2019) [↑](#endnote-ref-2)
4. Sparkes, J. & Holland, C. The rheological properties of native sericin. *Acta Biomater.* **69**, 234-242 (2018) [↑](#endnote-ref-3)
5. Asakura, T., Okushita, K. & Williamson, M. P. Analysis of the structure of Bombyx mori silk fibroin by NMR. *Macromolecules* **48**, 2345-2357 (2015) [↑](#endnote-ref-4)
6. Johnston, E. R., Miyagi, Y., Chuah, J. A., Numata, K. & Serban, M. A. Interplay between Silk Fibroin’s Structure and Its Adhesive Properties. *ACS Biomater. Sci. Eng.* **4**, 2815-2824 (2018) [↑](#endnote-ref-5)
7. Kwak, H. W., Ju, J. E., Shin, M., Holland, C. & Lee, K. H. Sericin Promotes Fibroin Silk i Stabilization Across a Phase-Separation. *Biomacromolecules* **18**, 2343-2349 (2017) [↑](#endnote-ref-6)
8. Holland, C., Terry, A. E., Porter, D. & Vollrath, F. Natural and unnatural silks. *Polymer (Guildf)*. **12**, 3388-3392 (2007) [↑](#endnote-ref-7)
